# Long-term Outcome for Two-Stage Palatal Closure With Different Timings for Hard Palate Surgery: Craniofacial Growth and Dental Arch Relation

**DOI:** 10.1177/10556656221140676

**Published:** 2023-01-03

**Authors:** Midia Najar Chalien, Hans Mark, Jan Lilja, Sara Rizell

**Affiliations:** 1 Clinic of Orthodontics, Public Dental Service, Region Västra Götaland, Gothenburg, Sweden; 2 Institute of Odontology, 70712 The Sahlgrenska Academy, University of Gothenburg, Gothenburg, Sweden; 3 Department of Plastic Surgery, Sahlgrenska University Hospital and Institute of Clinical Sciences at Sahlgrenska Academy, University of Gothenburg, Gothenburg, Sweden

**Keywords:** craniofacial growth, nonsyndromic clefting, dental arch, surgical technique

## Abstract

**Objective:**

The aim was to evaluate dental arch relation and craniofacial growth for individuals born with unilateral cleft lip and palate (UCLP), who had two-stage palatal surgery, with hard palate closure (HPC) at the ages of 3 or 8 years.

**Design:**

Longitudinal cohort study.

**Setting:**

Ceft lip and palate team in Gothenburg, Sweden.

**Patients:**

The inclusion criteria were nonsyndromic individuals born with complete UCLP who were consecutively operated according to two different surgical protocols; soft palate closure at 6 months followed by hard palate closure at the age of 3 years (HPC3) or at the age of 8 years (HPC8). In this evaluation, 28 individuals had HPC3 and 59 individuals had HPC8.

**Internventions:**

The main outcome, longitudinal series of dental casts and lateral radiographs from the ages of 5, 10, 16, and 19 years, were evaluated using GOSLON Yardstick and cephalometric analysis.

**Results:**

At the age of 10 years, 78% of the individuals with HPC3 demonstrated GOSLON scores of 1 and 2 and 86% in HPC8. At the age of 19 years, 54% of the individuals exhibited GOSLON scores of 1or 2 when compared with 74% in HPC8. A statistical significant difference for SNA was observed at the age of 5 years (*P*  *=*  .004), with a lower SNA in HPC3, but not at the ages of 10, 16 and 19 years. At the final age, SNA was 75.2° for HPC3 and 76.8° for HPC8.

**Conclusions:**

The decrease in age for HPC did not have an adverse effect on long-term dental arch relationship or craniofacial growth.

## Introduction

The surgical protocol for cleft lip and palate (CLP) remains a subject of controversy. However, an overall acceptance prevails for palatal surgery as one of the main reasons for restricted midfacial growth in individuals born with CLP.^1, 2^ A two-stage palatal closure, starting with soft and followed by hard palate, was proposed about a century ago by Gillies and Fry^3^ with the idea of minimizing the deteriorating effect from palatal surgery on maxillary growth. The technique was implemented by Schweckendiek and Doz^4^, closing the soft palate initially and delaying hard palate closure (HPC) until preadolescence, thus extending the period of less affected maxillary growth until development of palatal scar tissue. The mentioned technique has been reported with favorable midfacial growth^4–7^ but the findings are also contradicted.^8–10^ It is important to distinguish this surgical protocol from the two-stage procedure where the hard palate is closed initially followed by the soft palate.^11^

A two-stage surgical procedure with delayed HPC was introduced in 1975 by the Gothenburg CLP team and favorable long-term results regarding craniofacial growth have been exhibited.^12,13^ Also, long-term speech assessment revealed favorable results, although retracted oral articulation was observed until the age of 7 years due to the unoperated hard palate cleft.^14^ Thus, to decrease the impact from the residual hard palate cleft on articulation, the HPC was performed at the age of 3 years instead of 8 years.

Even though two-stage palatal closure has been questioned, it is a well-established technique among Europeancleft teams.^15^ A continuous evaluation of outcome is crucial to determine the quality of a surgical protocol. As CLP occurs relatively rarely, research with high evidence level such as randomized clinical trials (RCTs) are demanding to perform, why retrospective studies dominate.

However, inclusion of consecutively collected patients, followed longitudinally on long-term basis, enhance the level of scientific quality. Limited number of published studies present long-term longitudinal results for craniofacial growth and dental arch relationship, where the patients are followed from early childhood to late teens.^9–11,13,16–20^ The most common tool for the assessment of dental arch relation for individuals with unilateral cleft lip and palate (UCLP) is GOSLON Yardstick,^21^ which together with cephalometric analysis gives a comprehensive evaluation. Even though GOSLON scores and cephalometric outcome are correlated with respect to the sagittal relation of the maxilla and mandible,^22^ a combination of longitudinal cephalometric and GOSLON analysis will enhance the strength of the results. The aim was to compare dental arch relation and craniofacial growth for two consecutive series of individuals born with UCLP, who had HPC at the ages of 3 or 8 years. The null hypothesis was that there is no difference in dental arch relation and craniofacial growth for the two surgical protocols.

## Materials and Methods

### Patients

The inclusion criteria were nonsyndromic individuals born with complete UCLP (allowing a soft tissue bridge less than 5 mm), who had all surgery performed by the Gothenburg CLP team, and had finalized the follow-up according to the regional care program for CLP. In addition, only individuals who were consecutively operated according to following two surgical protocols were included. One surgical protocol included soft palate closure at 6 months of age followed by hard palate closure at the age of 3 years and (HPC3) secondary alveolar bone grafting (SABG) in mixed dentition.^23,24^ The second surgical protocol included soft palate closure at 6 months of age followed by hard palate closure together with SABG at the age of 8 years (HPC8).^13,23^

The HPC3 group consisted of 28 individuals (19 males and 9 females), born between 1993 and 1996, where 4 individuals exhibited a soft tissue bridge over the cleft. The HPC8 group consisted of 59 individuals (42 males and 17 females), born between 1980 and 1989, where 9 individuals exhibited a soft tissue bridge. The patients born between 1990 and 1992 did not have a defined timing for HPC and were therefore not included. Three individuals born between 1993 and 1996 were not included due to different HPC techniques. The treatment protocols included infant orthopedics with an acrylic plate. The patients received orthodontic expansion as required prior to SABG and comprehensive treatment was delivered in permanent dentition.

### GOSLON Yardstick

Dental casts (n  =  341) were collected at the ages of 5 (mean 5.0 years; range: 4.0-6.0), 10 (mean 10.0 years; range: 9.7-10.6), 16 (mean 16.1 years; range: 15.4-17.1), and 19 (mean 19.0 years; range: 17.0-19.9) years. The number of available cast models for each age is presented in Table 1. The dental arch relationship was assessed independently by 3 raters using the GOSLON Yardstick.^22^ Two of the raters were external and are thus not involved in Gothenburg CLP team. Before the assessment, the raters had a calibration course with one of the originators of the GOSLON Yardstick (Michael Mars) to reduce systematic bias.^22^ The assessors were blinded with respect to surgical protocol and GOSLON Yardstick reference models were available during the scoring. To calculate intra- and inter-rater reliability, 170 models were reassessed.

**Table 1.** GOSLON Scores of 1 to 5^a^ for HPC3 and HPC8 at the Ages of 5, 10, 16 and 19 Years^b^.

	5 years			10 years		16 years		19 years	
GOSLON score	HPC3 N(%)		HPC8 N(%)	HPC3 N(%)	HPC8 N(%)	HPC8 N(%)	HPC8 N(%)	HPC3 N(%)	HPC8 N(%)
1	6 (22)		21 (36)	4 (14)	10 (17)	2 (7)	5 (8)	0 (0)	2 (4)
2	12 (44)		28 (47)	18 (64)	41 (69)	17 (63)	38 (64)	14 (54)	39 (70)
3	8 (30)		9 (15)	6 (21)	7 (12)	6 (22)	12 (20)	10 (38)	10 (18)
4	1 (4)		1 (2)	0 (0)	1 (2)	2 (7)	3 (5)	2 (8)	5 (9)
5	0 (0)		0	0 (0)	0 (0)	0 (0)	1 (2)	0 (0)	0 (0)
Total	27 (100)	59 (100)		28 (100)	59 (100)	27 (100)	59 (100)	26 (100)	56 (100)
Mean GOSLON	2.1	1.8		2.1	2.0	2.3	2.3	2.5	2.3

^a^ GOSLON scores of 1 and 2 indicates the most favorable scores and GOSLON scores of 4 and 5 indicates the least favorable scores.

^b^ No statistical significant differences were found in the distribution of GOSLON scores between HPC3 and HPC8 using Mann–Whitney U-test. A *P* value <.05 was considered statistically significant.

Abbreviations: HPC3, hard palate closure at the age of 3 years; HPC8, hard palate closure at the age of 8 years.

### Lateral Cephalometrics

Lateral cephalograms (n  =  332) were obtained at the ages of 5 (mean 5.0 years; range: 4.0-6.0), 10 (mean 10.0 years; range: 9.7-10.6), 16 (mean 16.1 years; range:15.4-17.1), and 19 years (mean 19.0 years; range: 17.1-19.9). At the age of 5 years, 28 and 57 (HPC3/HPC8, respectively) lateral cephalograms were available and at the ages of 10, 16 and 19 years, 28 and 58, 24 and 59, and 24 and 54 cephalograms were available (HPC3/HPC8).

Standardized lateral cephalograms were taken with natural head position and the teeth in centric occlusion. The radiographs were traced by 1 observer (MNC) in the software program FACAD (Illexis, Sweden). The assessor was blinded with respect to surgical protocol. Twenty randomly selected lateral cephalograms were analyzed twice with an interval of 2 weeks to calculate the error of the measurement. Four angular measurements were chosen for the evaluation of craniofacial growth: SNA (anteroposterior maxillary position relative to the anterior cranial base), ANB (intermaxillary anteroposterior relation), NSL/ML (maxillary inclination relative to the anterior cranial base), and NL/ML (vertical intermaxillary relation).

The study was conducted according to the Helsinki Declarations and ethical approval was obtained from the Ethical Committee in Gothenburg (Dnr 1020-12).

### Statistical Analysis

The statistical analysis was carried out using the software SPSS (IBM, Inc.).

### GOSLON Yardstick

Coheńs weighted kappa statistics were used to evaluate the intra- and interrater reliability.

Nonparametric statistics, Mann–Whitney U-test was used to compare GOSLON scores between the 2 surgical protocols. A *P* value <.05 was considered statistically significant.

### Lateral Cephalometrics

The error of measurement was calculated according to Dahlberg.^25^

Generalized linear models (GLMs) fitted in the generalized estimating equation (GEE) framework were used to examine whether the surgical protocol had an impact on craniofacial growth, allowing for repeated measures on participants. Multivariable models using group, time as well as group and time interaction variables were fitted. Following estimation of the main model (ie, using CONTROLS as a reference group), the mean of SNA, ANB, NSL/NL, and NL/ML scores were estimated using SPSS's GENLIN function. In addition, pairwise comparisons of group mean at each time point were performed. The significance level of these pairwise comparisons was adjusted by Sidak's method.

## Results

### GOSLON Yardstick

**Figure 1.**
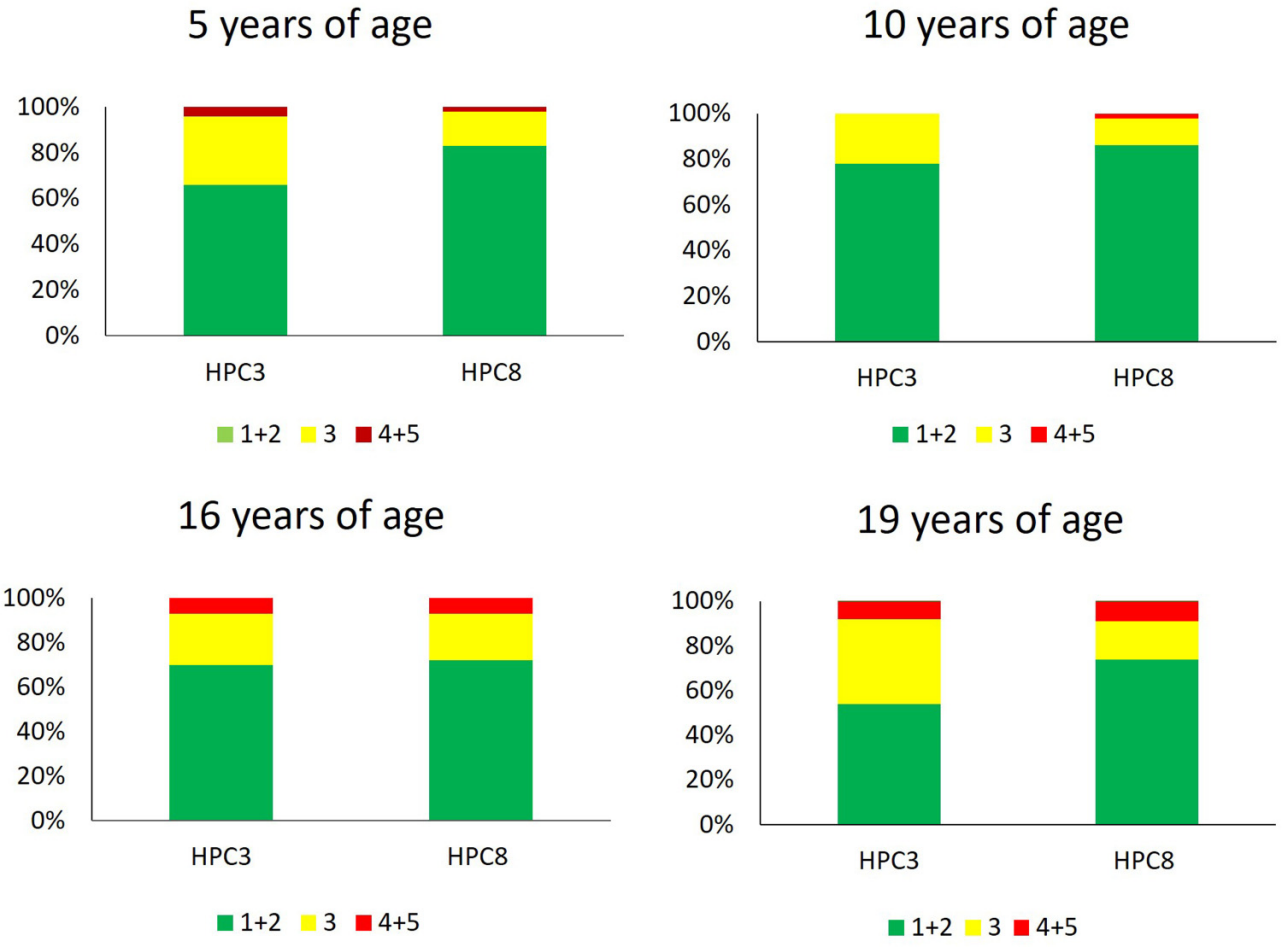
GOSLON scores for the two surgical protocols, with hard palate closure at the ages of 3 (HPC3) or 8 years (HPC8) and at the ages of 5, 10, 16, and 19 years. The green color indicates the most favorable scores (GOSLON scores of 1 and 2), yellow indicates GOSLON score of 3, and red indicates the least favorable scores (GOSLON scoresof 4 and 5). No statistical significant difference was found at the ages of 5, 10, 16, or 19 years between the 2 surgical protocols using Mann–Whitney *U*-test. A *P* value <.05 was considered statistically significant.

The intra-rater-reliability was found to be very good (0.79-0.84) while the inter-rater reliability was found to be good (0.6-0.78). The distribution of cast models, according to their given GOSLON score is shown in Table 1 and Figure 1, for HPC3 and HPC8. No statistical significant differences were observed between the two surgical protocols for any of the ages of 5, 10, 16, and 19 years. At the age of 5 years, 66% of the patients in HPC3 demonstrated favorable growth (GOSLON scores of 1 and 2) and 83% in HPC8. At the age of 10 years, an increased number of the patients in HPC3 (78%) demonstrated favorable growth (GOSLON scores of 1 and 2) and 86% in HPC8. In addition, at the age of 16 years, the portion of cast models revealed GOSLON scores of 1 and 2 decreased to 70% and 72% in HPC3 and HPC8, respectively, and finally 54% and 74% at the age of 19 years. The mean GOSLON score for the entire sample (5, 10, 16, and 19 years together) was found to be 2.3 for HPC3 and 2.1 for HPC8.

### Lateral Cephalogram

**Figure 2.**
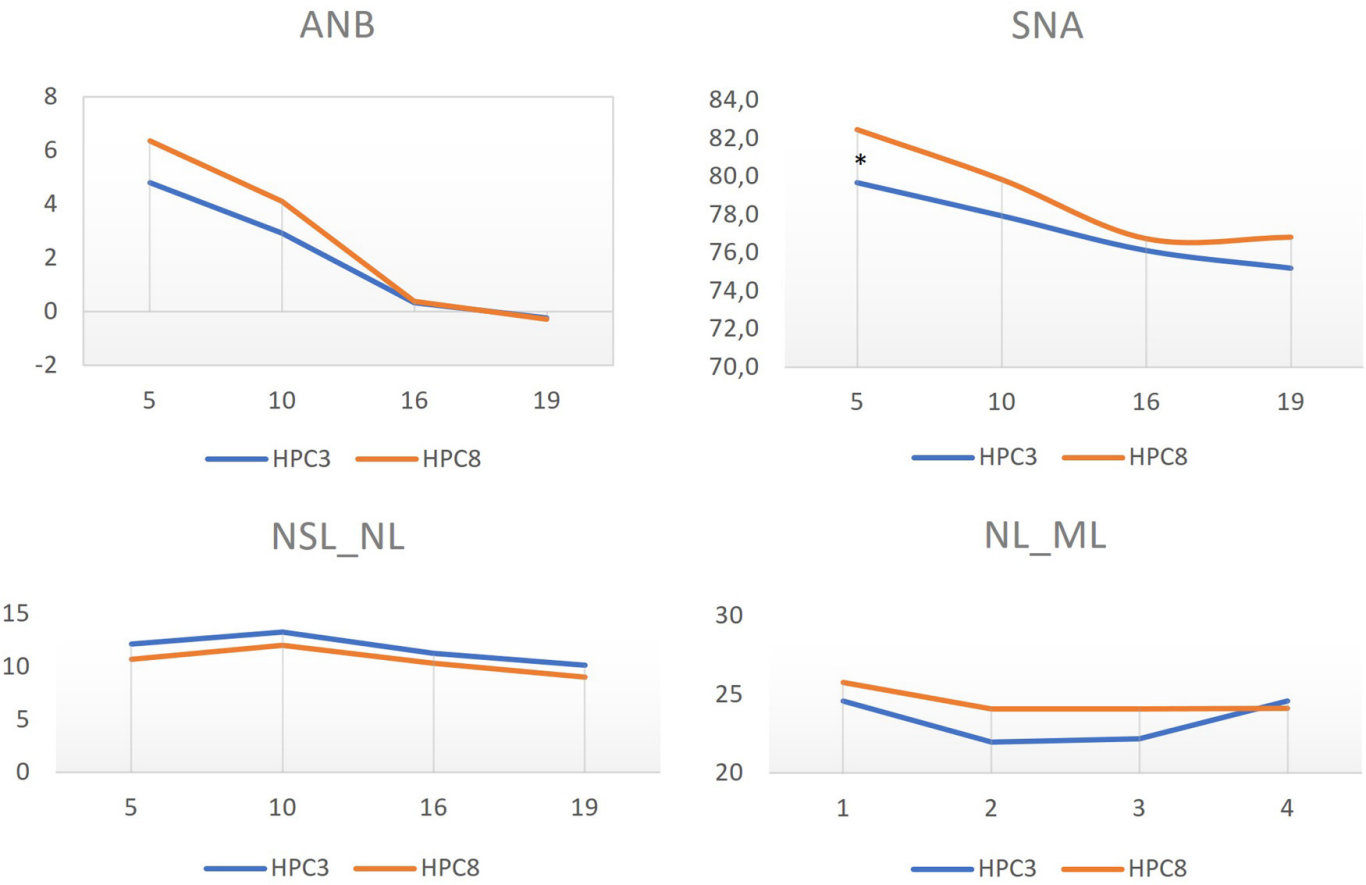
Cephalometric values for the two surgical protocols, with hard palate closure at the ages of 3 (HPC3) or 8 years (HPC8) and at the ages of 5, 10, 16, and 19 years. According to pairwise comparison using generalized estimating equation (GEE) with Sidak correction for multiple comparisons, a statistical significant difference was found between the groups only for the variable SNA at the age of 5 years (*P  *=*  *.004)*, with an increased SNA angle in the HPC8 group. A *P* value <.05 was considered statistically significant.

The error of measurement for the cephalometric analysis was calculated to 0.7 (SNA), 0.4 (ANB), 0.9 (NSL/NL), and 1.2° (NL/ML). A statistical significant difference for SNA was observed at the age of 5 years (*P*  *=*  .004) but not at the ages of 10, 16 and 19 years (Table 2,Figure 2). For ANB, NSL/NL, and NL/ML, no statistical significant difference was observed between HPC3 and HPC8 at any age (Table 2,Figure 2).

**Table 2.** Cephalometric Values for the 2 Surgical Protocols, HPC3 and HPC8, at the Ages of 5, 10, 16, and 19 Years.

Variable	Age	HPC3 Mean ± SE (range)	HPC8 Mean ± SE (range)	Sidak significance	95% Wald confidence interval for difference	
					Lower	Upper
SNA	5	79.7 ± 0.5 (78.6–80.7)	82.5 ± 0.5 (81.5–83.4)	0.004^a^	−5.08	−0.49
	10	77.9 ± 0.7 (6.4–79.4)	79.8 ± 0.4 (79.0–80.7)	0.5	−4.60	0.78
	16	76.1 ± 0.7 (74.7–77.5)	76.7 ± 0.4 (76.0–77.5)	1.0	−3.2	1.7
	19	75.2 ± 0.7 (73.7–76.6)	76.8 ± 0.4 (75.9–77.7)	0.83	−4.3	1.1
ANB	5	4.8 ± 0.4 (3.9–5.6)	6.3 ± 0.3 (5.8–6.9)	0.07	−3.2	0.06
	10	2.9 ± 0.4 (2.1–3.7)	4.1 ± 0.3 (3.5–4.7)	0.46	−2.8	0.4
	16	0.3 ± 0.6 ( − 0.8–1.5)	0.4 ± 0.3 ( − 0.2-0.9)	1.0	−2.1	1.9
	19	−0.2 ± 0.6 ( − 1.4–0.9)	−0.3 ± 0.3 ( − 0.9-0.4)	1.0	−2.0	2.1
NSL/NL	5	12.1 ± 0.6 (11.0–3.3)	10.7 ± 0.5 (9.7–11.7)	0.84	−0.9	3.8
	10	13.3 ± 0.6 (12.0–4.5)	12.0 ± 0.4 (11.2–12.9)	0.97	−1.2	3.7
	16	11.3 ± 0.8 (9.6–12.9)	10.4 ± 0.4 (9.5–11.2)	1.0	−2.0	3.8
	19	10.2 ± 0.9 (8.4–11.9)	9.0 ± 0.5 (8.0–10.0)	1.0	−2.1	4.4
NL/ML	5	24.6 ± 1.0 (22.7–6.4)	25.8 ± 0.6 (24.6–26.9)	1.0	−4.7	2.3
	10	22.0 ± 10 (20–23.9)	24.1 ± 0.6 (22.8–25.3)	0.88	−5.8	1.5
	16	22.2 ± 1.3 (19.6–4,8)	24.1 ± 0.7 (22.7–25.5)	0.99	−6.6	2.8
	19	24.6 ± 1.5 (21.7–27.4)	24.1 ± 0.8 (22.6–25.6)	1.0	−4.7	5.6

^a^ Statistical significant value with significance level set as *P* value < .05.

Abbreviations: ANB, A-point-nasion-B-point; HPC3, hard palate closure at the age of 3 years; HPC8, hard palate closure at the age of 8 years; ML, mandibular line; NSL, nasion-sella line; NL, nasal line; SNA, sella-nasion A point.

## Discussion

### Main Findings

The present study compared longitudinal long-term results between the surgical protocols HPC3 and HPC8. No difference for final outcome at the age of 19 years with respect to dental arch relationship and craniofacial growth was found between the two surgical methods. Except from maxillary prominence at the age of 5 years, dental arch relationship and craniofacial growth were corresponding during growth between the studied surgical protocols.

### Dental Arch Relationship

Only few studies present long-term GOSLON results that are obtained after cessation of growth from a consecutive series of patients.^10,13,18,26^ Despite final growth is the most important to evaluate, several centers present results for dental arch relation only until the age of 10 years.^27–30^ Our mean GOSLON at the age of 10 years was 2.1 and 2.0 for HPC3 and HPC8, respectively. The center in Nijmegen revealed a mean GOSLON score of 2.4 at the age of 9 years from a consecutive series of patients operated with two-stage palatal closure with HPC performed between the ages of 4 and 10 years.^29^ Furthermore, Fudalej et al. presented a comparable result (mean GOSLON score of 2.4), however, consecutively operated with a one-stage palatal closure.^30^ In our sample, the highest number of patients with favorable dental arch relationship was registered at the age of 10 years, where 78% of patients in HPC3 and 86% in HPC8 were rated with GOSLON scores of 1 or 2, while the proportion of patients considered to have excellent or very good dental arch relation decreased with age (Table 1). However, to interpret results from dental arch relation obtained at the age 10 years is hazardous since a portion of the cases assessed, might have experience from orthodontic treatment.^28,29^

At the age of 19 years, 54% of the patients ended up with GOSLON scores of 1 or 2 in HPC3 and 74% in HPC8 (Table 1). Furthermore, only 8% and 9% of the patients exhibited GOSLON scores of 4 or 5 in HPC3 and HPC8, respectively. In a comparison with long-term outcome from patients operated according to Veau–Wardill–Kilner palatoplasty (VWK), 56% were scored with GOSLON scores of 1 or 2 and 39% with GOSLON scores of 4 or 5.^18^ The number of individuals with favorable dental arch relationship coincides with the present even if the portion of patients with poor dental arch relation was increased in contrast to the Gothenburg sample. However, it is important to note that the cohort of VWK lost more than 50% of the participants until the age of 19 years. In the same article, patients operated with “minimal incision technique” reported a worse result, exhibiting GOSLON scores of 1 or 2 for only 34% of the cases. It is a challenging task to compare results between different studies depending on how they present their results. In the Eurocleft intercenter study, a modification of the GOSLON Yardstick was used which facilitates better GOSLON results.^10^ However, the mean GOSLON for the best Eurocleft centers was found between 1.7 and 2.2, at the age of 17 years, while we found a mean GOSLON score of 2.5 in HPC3 and 2.3 in HPC8, at the age of 19 years.^10^ Kappen et al., who followed the same example using the modified GOSLON Yardstick for the permanent dentition presented with 32% having a GOSLON score of 1 while 45% having GOSLON scores of 4 and 5 with a mean GOSLON score of 3.3.^26^

Our study revealed no difference for dental arch relation at any age between the two surgical protocols. A worsening of the dental arch relationship with time from the age of 5 to 19 years was noticed. This finding is though conflicting with studies where either stable or improved results were observed from childhood until ceased growth.^10,18^ However, the first mentioned study used a modified GOSLON Yardstick for the 19-year olds that facilitates better GOSLON scores.^10^ We could nevertheless see an initial improvement of GOSLON scores from the ages of 5 to 10 years, which confirm the previously presented results, where as many as 35.3% showed improvement of GOSLON category during this period.^31,32^ This enhancement might reflect that many of the patients by the age of 10 years have had alignment of the upper arch with correction of both the sagittal and transversal relation. The predictive validity of GOSLON Yardstick in growing individuals with UCLP has been questioned.^17,31^ Pegelow et al. found that less than 40% of 5-year olds with GOSLON scores of 4 or 5 and only 18% with a GOSLON score of 3 were predictable until the age of 19 years.^31^

### Craniofacial Growth

No statistical significant differences were observed between the two surgical protocols at the ages of 10, 16, and 19 years (Table 2). However, a statistical significant difference regarding maxillary prominence (SNA) was revealed between the two groups at the age of 5 years. The still unoperated in HPC8 might be a plausible explanation for the less restricted maxillary prominence since palatal surgery did not interfere with the growth site area.^33^ As mentioned, a corresponding difference for dental arch relationship was not found because the GOSLON Yardstick reflects the intermaxillary relation (ANB) more than maxillary prominence (SNA).

We found a mean SNA of 75.2 and 76.8°s (HPC3 and HPC8) at the age of 19 years, and similar results for maxillary prominence are presented by other centers.^9,34,35^ Enemark et al. presented a mean SNA of 72.9° in a cohort who had HPC at 10 weeks and palatoplasty with pushback procedure at 22 months.^16^ However, few previously published results emanate from a consecutive series of patients, which might be a reason for inclusion bias, leaving a question mark regarding the growth of those not being selected for a particular study. It is remarkable that centers with completely different surgical protocols demonstrate congruent craniofacial growth outcome which raise the question of impact from unknown factors than the surgical technique and timing.

A gradual reduction of SNA and ANB was seen for both HPC3 and HPC8 from the age of 5 to 19 years. The maxillary prominence (SNA) decreased with 4.5 and 5.6° for the two protocols, respectively, in contrast to noncleft population who exhibited an increase of 3.7°.^36^ The developmental changes in the present study are in accordance with findings from the mixed longitudinal study by Semb^11^ while a Danish sample of 57 UCLP patients showed an increased SNA reduction (7°) from the age of 5 to 21 years.^16^ The vertical intermaxillary relation (NL/ML) was relatively constant over time and in concordance with the results presented by Semb and Meazzini et al.^11,35^ However, for a noncleft population NL/ML decreased by around 6°, from 5 to 19 years of age.^36^

No patients in the present sample had orthognathic surgery before the final assessment, but one patient in HPC3, as well as five in HPC8 had a surgery after. However, two additional patients were planned for orthognathic surgery in the first group but due to the caries situation and denial of treatment, the surgery was canceled. It is important to point out that the frequency of patients having orthognathic surgery does not indicate the actual final growth outcome. Intercenter comparison of such numbers is hazardous due to different self-perception and subjective treatment need among patients or varying indication for surgery among centers.

### Factors Influencing Growth

Scar tissue as a result from cleft surgery is judged to be an underlaying cause for maxillary growth inhibition with varying impact depending on localization (ref). Lip surgery appears to have a moderate influence on maxillary growth, since 60% of patients with experience from lip closure only, exhibited GOSLON scores of 1 or 2 in comparison with 100% of the total unoperated individuals.^37^ However, the scarring developed in the hard palate is considered one of the principal causes for maxillary growth restriction and the impact from interference with the vomero-premaxillary suture is discussed.^33,38^ It is suggested that a later hard palate surgery, in a two-stage procedure is associated with better craniofacial growth and dental arch development.^7,29^ The craniofacial growth spurt occurs at the ages of 6 to 10 years in girls and 8 to 14 years in boys.^36^ Still, it appears that decreasing the age for HPC until the age of 3 years did not have any detrimental effect on growth compared with the previous surgical protocol. The residual hard palate cleft diminishes dramatically following soft palate closure and the most significant reduction in cleft width occurs within the first 18 months, indicating less reason for postponement of HPC as long as until the age of 8 years.^39^ A narrower residual cleft could, in theory, cause less impairment on the palatal tissue. Although HPC8 protocol was believed to cause limited growth restriction, it did however turn out to have a negative impact on speech, which was the main reason why Gothenburg CLP team brought the HPC timing forward from the ages of 3 to 8 years.^12,14^

### Methodological Considerations

One criterion for successful cleft palate surgery is favorable occlusion and several methods are described for evaluation of dental arch relationship for individuals with CLP.^21^ The GOSLON Yardstick was chosen for assessment since it is used worldwide, which facilitates comparison of results.^21^ In addition, the GOSLON Yardstick has been shown to take shorter time to use in comparison with the 5-year-olds’ index (5YO), Modified Huddart/Bodenham scoring system (MHB), and Eurocran Index, even if MHB exhibited better intra- and inter-examiner reliability.^21^ Since GOSLON Yardstick requires subjective judgment from a clinically experienced examiner, a calibration course is needed, in contrary to MHB which also provides more site-specific information of the dental arch relation deficiency. Furthermore, MHB does not take into account plausible orthodontic camouflage of skeletal discrepancies and the sum of scores are difficult to interpret into clinical reality, while the GOSLON scores are applicable for clinical guiding. Although the GOSLON Yardstick was not primarily developed for evaluation of dental arch relationship in the primary dentition, agreement between the 2 indices (GOSLON yardstick and 5YO) has been found, why GOSLON Yardstick was chosen as measure for all the age groups in the present study.^22,31,40^

Presenting the GOSLON scores into 3 groups (1  +  2, 3, and 4  +  5) is questioned due to an enhancement of the coarseness of the yardstick. However, to facilitate intercenter comparison and interpretation of the results, we chose to categorize the scores (Figure 1) but the full distribution of all the scores although Table 1.^29^ The GOSLON Yardstick renders categorical data, and presentation of mean scores is therefore a subject of controversy. However, since it is widely used, we also chose to present the mean value to enable comparison with other centers.

### Strengths and Limitations

The principal strength is the longitudinal and long-term approach, where final outcome was assessed after cessation of growth. Even if the study design was retrospective the surgical as well as the follow-up protocol were determined prospectively, and documentation was planned at the ages of 5, 10, 16, and 19 years as part of the care program for children with CLP. In addition, a consecutive sample collection decreased the inclusion bias even if a randomized inclusion would have rendered higher scientific evidence. One additional strength is that speech results for the the groups HPC3 and HPC8 are available, and an improvement of the oral articulation was found for HPC3 (unpublished data).

Beside the surgical technique and timing, a correlation between experience of the surgeon and craniofacial growth outcome is discussed.^41,42^ In our sample, the same surgical teams, including threee surgeons, were operating the patients in the two groups. However, one high-volume surgeon performed the majority of the surgeries during both time periods (HPC3 and HPC8).

The number of patients in HPC3 was rather on the small side. However, the sample was consecutively collected and from 1997 the Gothenburg CLP team were recruiting participants for the multicenter Scandcleft RCT, why no additional patients were to be included.^43^ The two surgical protocols showed an uneven gender distribution with an increased proportion of males in the HPC8 group. Females are expected to discontinue growth approximately at the age of 16 years and males around the age of 18 years.^36^ Even if no statistical significant differences were found at any age except from the age of 5 years, it is important to keep in mind that such a disproportion might bias the outcome.

At the age of 19 years, 2 patients in HPC3 and 3 in HPC8 dropped out from the GOSLON assessment. The drop out data was caused by patients failing to arrive or registrations of poor quality. To reject the question if the dropouts have biased the results, their GOSLON scores at the age of 16 years were analyzed. In HPC3, the mean GOSLON score for the dropouts was 2.2 and 2.1 in HPC8, why the impact from the lost data was considered to be less plausible.

## Conclusion

The earlier timing of HPC, from the ages of 8 to 3 years in the two surgical protocols, did not have an adverse effect on neither dental arch relationship nor craniofacial growth. The null hypothesis was therefore accepted at the 5% significance level. Both the protocols, with two-stage HPC, were considered having corresponding favorable long-term outcome for dental arch relationship and craniofacial growth. An improvement of the compromised oral articulation has been found, after decreasing the age of HPC to the age of 3 years, which was one the main reasons for changing the surgical protocol (unpublished data). Therefore, earlier HPC is considered justified for long-term craniofacial growth, dental arch relationship, and oral articulation outcome.

The present results were based on a consecutive series of patients, who were followed from early childhood to cessation of growth. Evaluation of craniofacial growth outcome after termination of growth is crucial, when searching for the optimal timing and technique for primary cleft surgery. Additional longitudinal long-term studies are needed to be able to answer the persisting question of which surgical method to choose.
